# 917. Infectious Diseases Consult Improves Management of Candidemia

**DOI:** 10.1093/ofid/ofac492.762

**Published:** 2022-12-15

**Authors:** Nick Stornelli, Nathan Everson, Nathan Everson, Nathan Everson, Lauren McDaniel, Melissa White

**Affiliations:** Carilion Clinic, Roanoke, Virginia; Carilion Clinic, Roanoke, Virginia; Carilion Clinic, Roanoke, Virginia; Carilion Clinic, Roanoke, Virginia; Carilion Clinic, Roanoke, Virginia; Penn Medicine: Lancaster General Health, Lancaster, Pennsylvania

## Abstract

**Background:**

Infectious Diseases consultations (IDC) have been associated with improved outcomes in multiple disease states with high morbidity/mortality like invasive candidiasis. This study evaluated management of candidemia at Carilion Clinic in patient with and without IDC.

**Methods:**

A retrospective, observational cohort study was performed among hospitalized patients with candidemia who either received or did not receive an IDC between January 2014 and August 2020. Patients included had Candida species from at least one blood culture. Exclusions were death or transfer to a palliative care plan within 48 hours of culture, cultures from an outside facility, and patients that were pregnant or incarcerated. The primary outcome was a composite of all-cause mortality and recurrent candidemia within 90 days. Secondary outcomes included compliance with candidemia management bundle items such as echocardiography, ophthalmologic evaluation, receipt of susceptible antifungal, source control and repeated blood cultures.

**Results:**

A total of 194 patients were included in the study. The population was 50.3% male sex and mean ± standard deviation age was 57.8 ± 17.2 years. In the study timeframe, 131 patients received an IDC while 62 did not receive an IDC. ICU level of care, vasopressor requirement and Candida Score ≥ 3 were all similar between the two groups. Candida albicans was the most common species in IDC and non-IDC groups (48.1% vs. 41.1%). Empiric therapy was more commonly an echinocandin in 138/194 (71.1%) and definitive therapy was fluconazole in 117/194 (60.3%). An improved primary composite outcome was observed in the IDC group (34.9% vs. 21.4%, p = 0.043). Patients receiving IDC had a higher rate of repeated cultures (100% vs. 92%) and ophthalmologic examination (54.2% vs. 20.6%). More patients in the IDC group were found to have infective endocarditis (10.7% vs. 1.6%, p = 0.04) as they were more likely to have echocardiographic imaging (86.2% vs. 57.1%, p< 0.0001). The median time to susceptible antifungal was 43 (27-61) hours without ICD and 35 (27-52) hours compared in those with IDC.

**Conclusion:**

A more comprehensive workup by IDC was associated with more identification of endocarditis and less recurrence of candidemia within 90 days.

**Disclosures:**

**Nathan Everson, PharmD, BCIDP**, Merck: Advisor/Consultant|Merck: Grant/Research Support **Nathan Everson, PharmD, BCIDP**, Merck: Advisor/Consultant|Merck: Grant/Research Support **Nathan Everson, PharmD, BCIDP**, Merck: Advisor/Consultant|Merck: Grant/Research Support **Lauren McDaniel, PharmD, BCIDP**, Merck: Grant/Research Support.

